# General synthetic strategy for regioselective ultrafast formation of disulfide bonds in peptides and proteins

**DOI:** 10.1038/s41467-021-21209-0

**Published:** 2021-02-08

**Authors:** Shay Laps, Fatima Atamleh, Guy Kamnesky, Hao Sun, Ashraf Brik

**Affiliations:** Schulich Faculty of Chemistry, Technion-Israel Institute of Technology, Haifa, Israel

**Keywords:** Chemical synthesis, Synthetic chemistry methodology, Photochemistry

## Abstract

Despite six decades of efforts to synthesize peptides and proteins bearing multiple disulfide bonds, this synthetic challenge remains an unsolved problem in most targets (e.g., knotted mini proteins). Here we show a de novo general synthetic strategy for the ultrafast, high-yielding formation of two and three disulfide bonds in peptides and proteins. We develop an approach based on the combination of a small molecule, ultraviolet-light, and palladium for chemo- and regio-selective activation of cysteine, which enables the one-pot formation of multiple disulfide bonds in various peptides and proteins. We prepare bioactive targets of high therapeutic potential, including conotoxin, RANTES, EETI-II, and plectasin peptides and the linaclotide drug. We anticipate that this strategy will be a game-changer in preparing millions of inaccessible targets for drug discovery.

## Abstract (web summary)

Synthesis of peptides and proteins containing multiple disulfide bonds is challenging, limiting the elucidation of their biological functions. Here, the authors report a general synthetic strategy for fast formation of two and three disulfide bonds in peptides and proteins, and apply it to prepare several therapeutically important peptides.

## Introduction

After production in the ribosome, most proteins undergo further maturation through covalent modifications, which alter their structure, localization or function and aberrations in these steps are associated with numerous diseases^1^. While unmodified proteins can be readily obtained by biological expression, the preparation of posttranslationally modified proteins remains challenging, increasing demand for their chemical synthesis^1,2^.

Disulfide bonds, one of the most widespread covalent modifications, influences the three-dimensional architecture and function of peptides and proteins exist in many target of therapeutic interest such as in the insulin hormone, the coronavirus (SARS-CoV-2) spike protein^3,4^, and in natural libraries of toxin peptides among others. Unfortunately, performing studies with isolated or biologically expressed peptides and proteins that contain disulfide bonds, is time-consuming and often impractical and even impossible for various targets, driving the great interest in finding approaches for their efficient synthesis. Yet, their chemical synthesis remains as a long standing synthetic challenge for over six decades^5–8^, leaving various potential therapeutic targets inaccessible^3,6,7,9^.

**Fig. 1:**
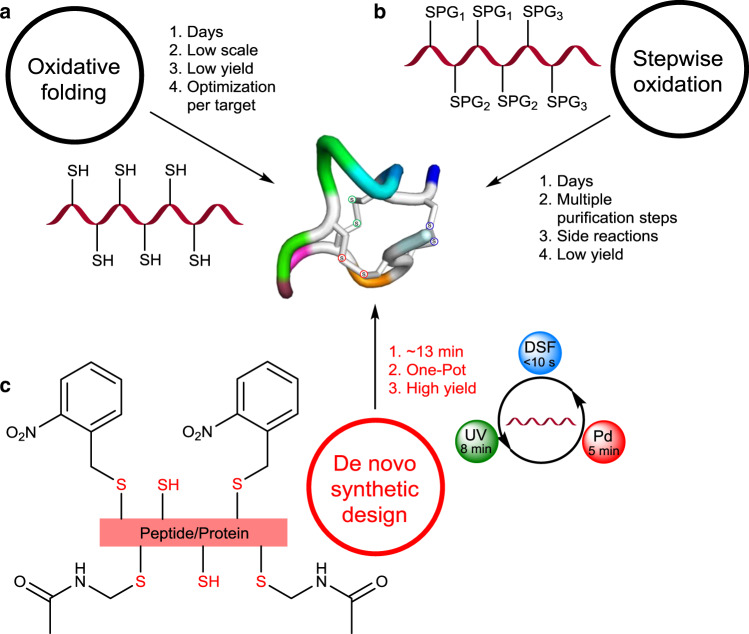
Prior strategies and our synthetic design for the synthesis of peptides and proteins with three disulfide bonds. **a** Oxidative folding under thermodynamic control, main limitations. **b** Stepwise deprotection and oxidation strategy, main limitations. **c** Ultrafast high-yielding formation of multiple disulfide bonds via our strategy (PG is abbreviation for protecting group, UV is abbreviation for ultraviolet-light and DSF is abbreviation for disulfiram).

This synthetic challenge led to exploration of two main strategies; (1) oxidative folding, which is common and depends on the protein folding pathway conferring the correct disulfide connectivities under thermodynamic control (e.g., the correct isomer out of the 75 possibilities for three disulfide bonds). This strategy suffers from various drawbacks such as long reaction times (up to several days), low yields or lack of success in many targets, probably due to the accumulation of kinetically trapped folded intermediate (dead-ends) (Fig. 1a)^3,6,7,10^. (2) The second strategy is based on stepwise formation of disulfide bonds employing orthogonally protected cysteine (Cys). Here, the removal of protecting groups (PGs) can lead to significant irreversible amino acids (AAs) side reactions and disulfide bonds isomerization, in addition to the lengthy process (several days) due to long reactions time and the necessity for multiple purification steps, which also leads to significant loss of material (Fig. 1b)^11–14^.

Here we show, an effective synthetic approach based on a general design for the synthesis of peptides and proteins containing disulfide bonds. Our strategy relies on small molecule activation of the Cys side chain via the disulfiram (DSF) and ultraviolet (UV) light/Pd chemoselective chemistries for one-pot and ultrafast formation of two or three disulfide bonds (Fig. 1c). We demonstrate the power and potential of this strategy for the preparation of various challenging targets in the efficient chemical syntheses of α-conotoxin SI peptide, RANTES protein from the chemokine family, EETI-II trypsin inhibitor knotted mini protein, plectasin antimicrobial peptide and the linaclotide peptide drug.

## Results and discussion

We set out to design an effective set of sequential reactions guided by the following considerations. First, the synthesis must be accomplished rapidly to eliminate accumulation of side products and/or reshuffling. Second, the chemistry should be carried out under aqueous denaturation conditions to avoid aggregation and kinetically trapped folding intermediates. Third, all steps must be performed in a one-pot operation for efficient synthesis^3,9^.

### Devising efficient synthesis of α-conotoxin and RANTES with two disulfide bonds

As a model system we chose the well characterized and studied α-conotoxin SI peptide, composed of 13 AAs and having two disulfide bonds between Cys (2&7) and Cys (3&13) that induce the 3_10_ helix shape^15^. This peptide belongs to the Conus venoms conotoxin family— bearing multiple disulfide bonds that stabilize their compact structures. Such properties are responsible for their potency against voltage-gated ion channels, G-protein-coupled receptors and neurotransmitter transporters, making them attractive leads for drug development^6^.

Direct disulfide bond formation from a protected Cys precursor has been challenging due to several side reactions and/or to reshuffling when multiple disulfide bonds exist. A recent study has shown that Pd and diethyldithiocarbamate (DTC) shown an effective removal of acetamidomethyl (Acm) PG and the direct formation of a single disulfide bond in peptides with no side reaction^16^. Yet, avoiding reshuffling remains a significant challenge in exploring these reagents in the efficient synthesis of peptides and proteins with multiple disulfide bonds. Nevertheless, we speculated that the Pd thiophilic nature and disulfiram (DSF), the oxidized and possibly more reactive form of DTC, could be employed to tackle this synthetic problem.

**Fig. 2:**
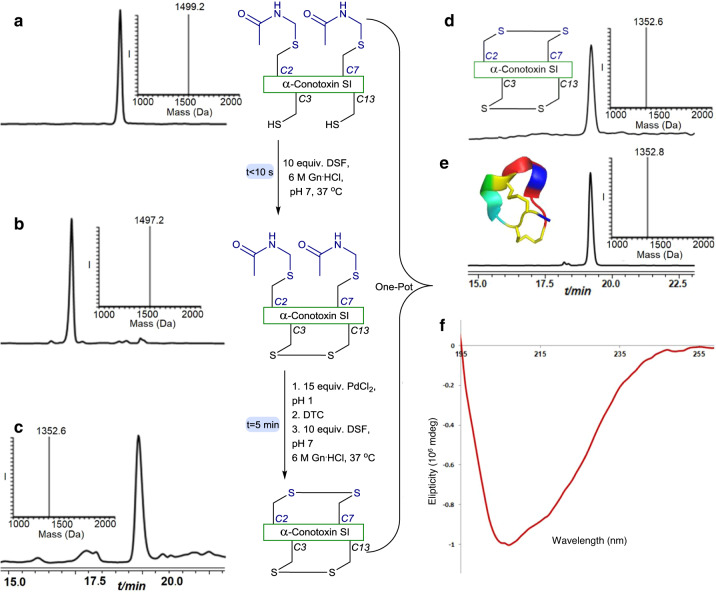
Two disulfide bonds formation in α-conotoxin. **a** HPLC-ESI MS analyses: Reaction at time zero, the main peak corresponds to reduced α-conotoxin peptide modified with two Acm groups at Cys (2&7) with the observed mass 1499.2 ± 0.1 Da, calcd. 1499.6 Da (average isotopes). **b** Reaction after 10 s: the main peak corresponds to α-conotoxin peptide bearing one disulfide bond modified with two Acm groups at Cys (2&7) with the observed mass 1497.2 ± 0.1 Da, calcd. 1497.6 Da (average isotopes). **c** In situ addition of 15 equiv. PdCl_2_ for 5 min followed by DTC/DSF treatment: the main peak corresponds to α-conotoxin peptide bearing two disulfide bonds with the observed mass 1352.6 ± 0.1 Da, calcd. 1353.6 Da (average isotopes). **d** Purification and folding: the main peak corresponds to α-conotoxin peptide bearing two disulfide bonds with the observed mass 1352.6 ± 0.1 Da, calcd. 1353.6 Da (average isotopes). **e** commercially available native α-conotoxin with the observed mass 1352.8 ± 0.1 Da, calcd. 1353.6 Da (average isotopes) (**f**) synthetic α-conotoxin CD spectrum.

To examine this notion, we prepared the linear α-conotoxin SI peptide by solid phase peptide synthesis (SPPS), with two Cys (3&13) protected with the trityl PG to afford the free S–H of these Cys residues after trifluoroacetic acid (TFA) cleavage, and two additionally modified Cys (2&7) with the (Acm) moiety, (Supplementary Fig. 1). Exposing α-conotoxin SI to DSF at 37 °C, pH 7, led to the immediate formation of the first disulfide bond between Cys (3&13), as detected by HPLC-ESI MS. To form the second bond between Cys (2&7) we added, in situ, PdCl_2_ for 5 min in pH 1. Notably carrying out this step at higher pHs led to significant disulfide reshuffling. Although the exact pathway of the reshuffling step still unclear, this on the other hand highlights the challenge in this synthetic endeavor. Subsequently, we added DTC, as a Pd scavenger^16^, and a fresh amount of DSF followed by adjustment of the reaction to pH 7. The second disulfide bond was formed immediately as shown by HPLC-MS analysis and we isolated the desired product in 48% yield (Fig. 2a–c). Circular dichroism (CD) measurements showed the expected signature of the 3_10_ helix native isomer (Supplementary Fig. 2 and Fig. 2f) confirming the formation of the native α-conotoxin SI peptide with the correct disulfide bonds^15^. In addition, the chromatographic retention time of our synthetic product matched perfectly to the commercially available peptide (Fig. 2d, e, respectively).

**Fig. 3:**
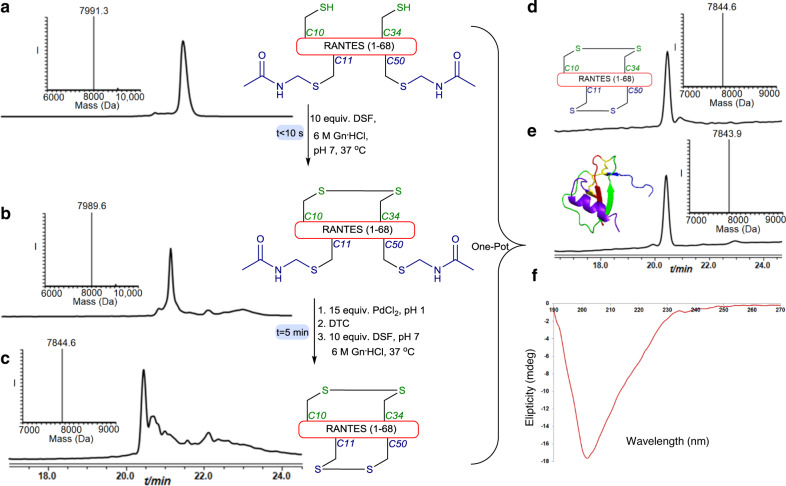
RANTES synthesis. **a** HPLC-ESI MS analyses: Reaction at time zero, the main peak corresponds to reduced RANTES modified with two Acm groups and two free Cys with the observed mass 7991.3 ± 0.1 Da, calcd. 7992.0 Da (average isotopes). **b** Reaction after 1 min: the main peak corresponds to RANTES bearing one disulfide bond and modified with two Acm with the observed mass 7989.6 ± 0.3 Da, calcd. 7990.0 Da (average isotopes). **c** Reaction after 5 min:HPLC-ESI MS, the main peak corresponds to RANTES bearing two disulfide bonds with the observed mass 7844.6 ± 0.1 Da, calcd. 7846.0 Da (average isotopes). **d** Purification and folding: the main peak corresponds to RANTES bearing two disulfide bonds with the observed mass 7844.7 ± 0.1 Da, calcd. 7846.0 Da (average isotopes). **e** commercially available native RANTES: analyses with the observed mass 7843.9 ± 0.1 Da, calcd. 7846.0 Da (average isotopes). **f** CD spectrum of the synthetic RANTES.

These encouraging results motivated us to examine our set of conditions in more challenging synthetic systems. Therefore, we chose the RANTES chemokine protein from the cytokine family that directs trafficking of leukocytes during inflammation^17^. We prepared this protein, composed of 68 AAs and two disulfide bonds between Cys (10&34) and Cys (11&50)^18^, via SPPS with Cys 10 and 34 in the free form, and Cys 11 and 50 modified with the Acm (Supplementary Fig. 3). Applying our developed synthesis afforded the native protein with the correct disulfides within 5 min, which was isolated in 35% yield for the two oxidation steps (Fig. 3a–c). The chromatographic retention time of the product matched perfectly with the commercially available protein and the CD signature of the correct isomer folding was detected (Fig. 3d–f).

#### One-pot formation of three disulfide bonds: plectasin and linaclotide syntheses

Next we sought to develop conditions for formation of three disulfide bonds in a one-pot operation taking advantage of the above describe conditions. We searched for possible orthogonal chemistry with the Pd and DSF before or after the Acm removal and in presence of at least a single disulfide bond. Therefore, we envisioned a possible pathway involving Pd/light irradiation, as disulfide bond is known to be stable to light irradiation^19^. To evaluate this notion, we synthesized the α-conotoxin peptide bearing two Cys (3&13) modified with 2-nitrobenzyl (NBzl) and other two Cys (2&7) modified with Acm. We examined the use of the NBzl photosensitive PG under exposure to our conditions because of its relatively easy incorporation to the Cys side chains and its high stability in SPPS^20,21^. To our pleasant surprise when the α-conotoxin peptide was exposed to UV light in presence of DSF at pH 7, a fast (within 8 min) and simultaneous decaging and selective disulfide formation, was observed without affecting the Acm (Supplementary Fig. 4). Notably, this decaging step was expedited by the disulfide formation in presence of DSF^20^. Following the UV step, we then added Pd^II^ and DTC/DSF, which led to the formation of the desired product within only 5 min without any detectable side products (Supplementary Fig. 4).

Based on these results with the model peptide, we designed the following synthetic one pot operation for the synthesis of peptides and proteins with three disulfide bonds, which includes; (1) DSF mediated formation of the first disulfide bond (<10 s), (2) UV light exposure (~8 min) to form the second disulfide bond from a Cys (NBzl) precursor, (3) Pd^II^ DTC/DSF mixture mediated third disulfide bond formation from a Cys (Acm) precursor (~5 min).

**Fig. 4:**
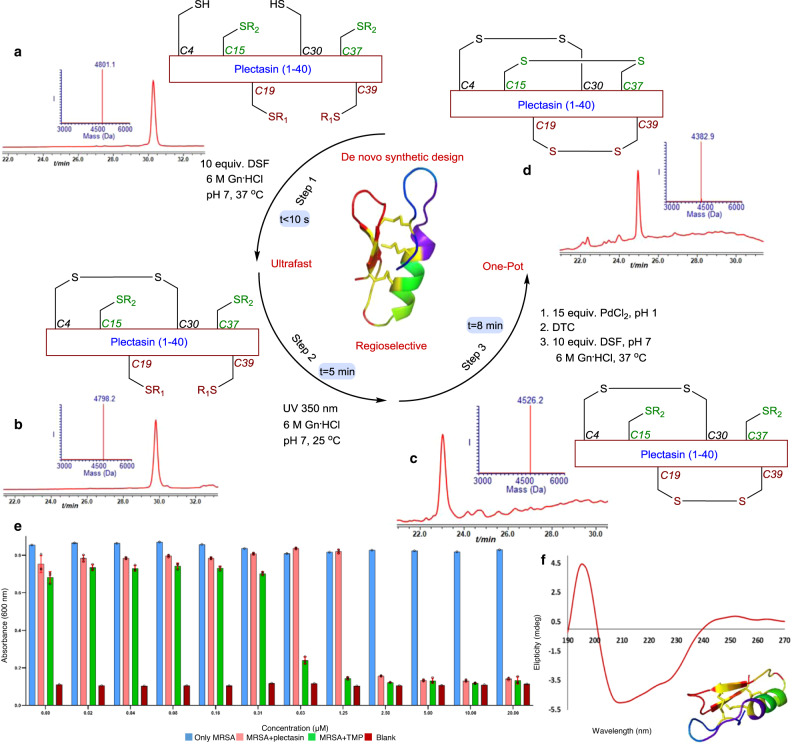
Synthesis of plectasin employing our synthetic design. **a** HPLC-ESI MS analyses Reaction at time zero, the main peak corresponds to reduced plectasin with the observed mass 4801.1 ± 0.1 Da, calcd. 4801.0 Da (average isotopes). **b** Reaction after 10 s: the main peak corresponds to plectasin with single disulfide bond, with the observed mass 4798.2 ± 0.1 Da, calcd. 4798.0 Da (average isotopes). **c** Reaction after 8 min, the main peak corresponds to plectasin with two disulfide bonds with the observed mass 4526.2 ± 0.2 Da, calcd. 4527.0 Da (average isotopes). **d** Reaction after 13 min, the main peak corresponds to fully oxidized plectasin with the observed mass 4382.9 ± 0.1 Da, calcd. 4383.0 Da (average isotopes). **e** Plectasin activity assay: absorbance monitoring at 600 nm for methicillin resistant staphylococcus aureus (MRSA) growth after 7 h in the absence (in light blue) and presence (in light red) of synthetic plectasin and MRSA in the presence of trimethoprim (TMP) (in gray). LB was used as medium in the assay (in dark red). Data are represented as mean ± SD, *n* = 3 biologically independent samples, error bars represent the SD. IC50 ~1.5–2 µM. R_1_ = NBzl, R_2_ = Acm. **f** Plectasin CD.

**Fig. 5:**
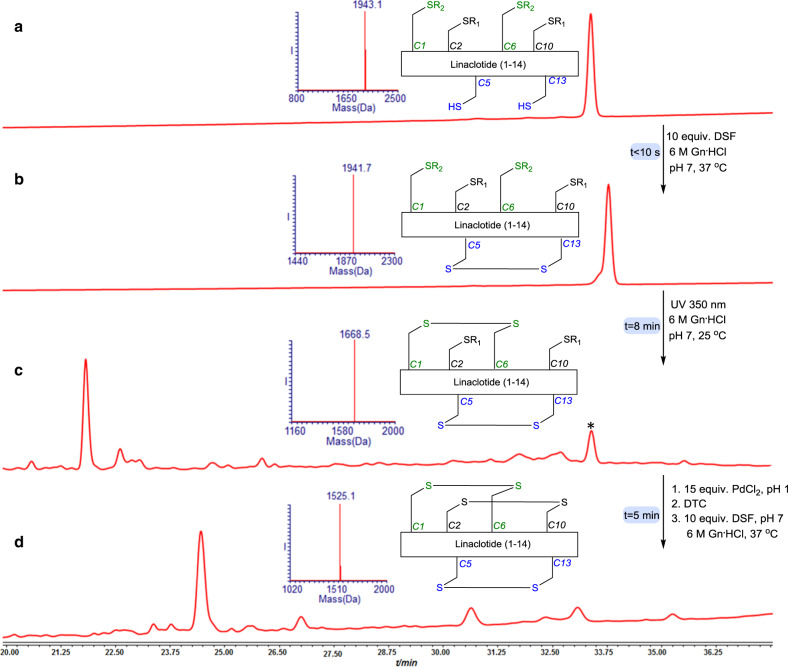
Linaclotide synthesis. **a** HPLC-ESI MS analyses: Reaction at time zero, the main peak corresponds to reduced Linaclotide with the observed mass 1943.1 ± 0.1 Da, calcd. 1944.2 Da (average isotopes). **b** Reaction after 10 s, the main peak corresponds to Linaclotide with single disulfide bond with the observed mass 1941.7. ± 0.1 Da, calcd. 1941.1 Da (average isotopes). **c** Reaction after 8 min: the main peak corresponds to Linaclotide with two disulfide bonds with the observed mass 1668.5 ± 0.2 Da, calcd. 1669.1 Da (average isotopes). **d** Reaction after 5 min: the main peak corresponds to Linaclotide with the three disulfide bonds with the observed mass 1525.1 ± 0.1 Da, calcd. 1525.1 Da (average isotopes). R_1_ = NBzl, R_2_ = Acm. *non peptide mass correspondence to small molecule decomposition.

To evaluate the practicability of this design, we chose the plectasin peptide, known as a fungal defensin potently active against drug-resistant Gram-positive bacteria (e.g., streptococci)^22^. This peptide is made of 40 AAs with three disulfide bonds between Cys (4&30), Cys (19&39), Cys (15&37) and shares structural features with the other members of this family. The linear polypeptide was assembled via SPPS protocol, bearing two free Cys (4&30), two Cys modified with NBzl (19&39) and two Cys modified by Acm (15&37) (Supplementary Fig. 5). The peptide was subjected to our synthetic design and a single product was formed within 13 min and was isolated in 43% yield (Fig. 4a–d). The folded protein eluted with the same retention time as the product obtained via oxidative folding following previously used protocols (Supplementary Fig. 6), exhibited the expected biological antibiotic activity (Fig. 4e) and showed the expected CD signature of the natural isomer laddered shape and (Fig. 4f)^23^. Using our protocol we have also prepared the linaclotide drug for chronic constipation and irritable bowel syndrome, made of a 14 AAs peptide and bearing three disulfide bonds (Fig. 5 and Supplementary Figs. 7, 8)^3^.

#### Synthesis of EETI-II knotted mini protein

**Fig. 6:**
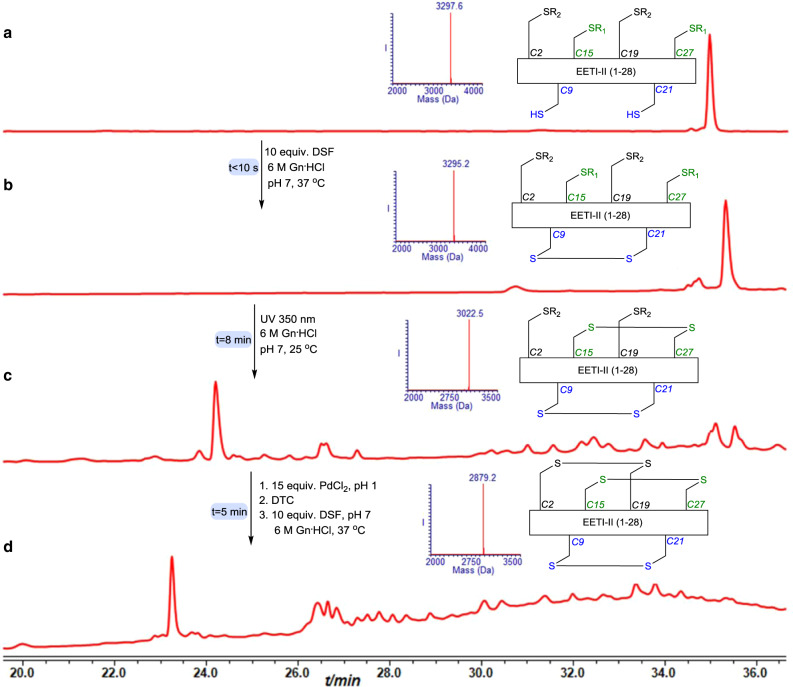
EETI-II synthesis. **a** HPLC-ESI MS analyses: Reaction at time zero, the main peak corresponds to reduced EETI-II with the observed mass 3297.6 ± 0.1 Da, calcd. 3297.4 Da (average isotopes). **b** Reaction after 10 s, the main peak corresponds to EETI-II with single disulfide bond with the observed mass 3295.2 ± 0.1 Da, calcd. 3295.4 Da (average isotopes). **c** Reaction after 8 min: the main peak corresponds to EETI-II with two disulfide bonds with the observed mass 3022.5 ± 0.2 Da, calcd. 3023.4 Da (average isotopes). **d** Reaction after 13 min: the main peak corresponds to EETI-II with three disulfide bonds with the observed mass 2879.2 ± 0.1 Da, calcd. 2879.4 Da (average isotopes). R_1_ = NBzl, R_2_ = Acm.

**Fig. 7:**
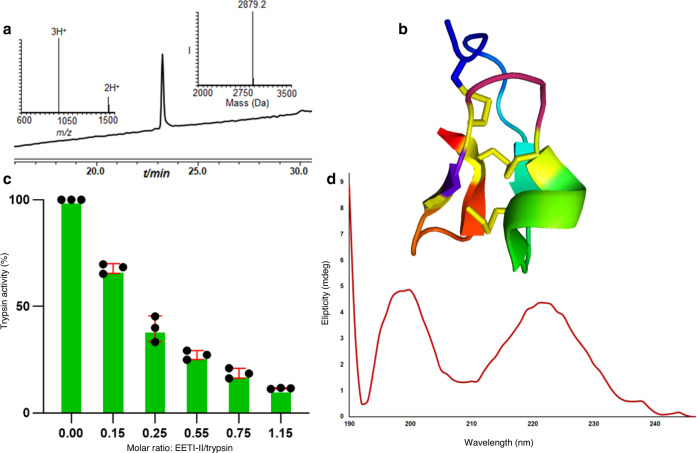
Synthetic EETI-II characterization. **a** HPLC-ESI MS analyses of purified and folded EETI:, the main peak corresponds to EETI-II bearing three disulfide bonds with the observed mass 2879.2 ± 0.1 Da, calcd. 2879.4 Da (average isotopes). **b** EETI-II structure (PDB). **c** Trypsin inhibition biological activity assay. Data are represented as mean ± SD, *n* = 3 biologically independent samples, error bars represent the SD. **d** CD spectrum of synthetic EETI-II.

With these results in hands, we decided to examine our synthetic design on a more challenging example from the knotted protein family. The synthesis of molecular knots has been a challenging problem that has spurred synthetic efforts for the past 50 years^24^. In particular the synthesis of Cys knot structural motif that has exceptional effects on peptide/protein chemo, mechano and proteolytic stability has remained difficult to the point of seemingly impossible^7^. We chose as a representative example the well-studied EETI-II mini protein, composed of 28-residues and belonging to the squash family of trypsin inhibitors, which has never been

prepared using the stepwise approach^25,26^. Using native chemical ligation in solution or on resin coupled with oxidative folding the protein was successfully prepared. Yet, the oxidative folding step took up to 10 days for completion^25^. To examine our strategy we used SPPS to afford the linear peptide bearing free Cys (9&21), Cys (15&27) modified with NBzl and Cys (2&19) modified with the Acm (Supplementary Fig. 9). Applying our synthetic three steps operation, the protein was formed with native disulfide connectivities within 13 min (Fig. 6 and Supplementary Fig. 10). The product was isolated in 32% yield and showed the native biological activity^25^ in addition to the expected unique CD signature (Fig. 7).

In summary, we introduced here a straightforward synthetic scheme applying activation of the Cys side chain by DSF, UV light, and Pd for chemoselective and regioselective formation of multiple disulfide bonds, tackling a decades old standing problem in peptide and protein chemistry. This efficient three step, one-pot operation was demonstrated in the syntheses of representative examples from various large peptide/protein families. This strategy is currently restricted to three disulfide bonds and the synthesis of targets with more than three disulfide bonds will require the development of additional chemistry for chemoselective and regioselective disulfide bond formation. We anticipate that this synthetic design will pave the way for the synthesis of currently inaccessible targets due to its excellent efficiency and simplicity. Moreover, we envision with the recent technological progress in SPPS employing flow^27–29^ and robotic chemistry^30^, the generation of combinatorial libraries of peptides and proteins bearing multiple disulfide bonds for drug discovery and various other applications.

## Methods

The following stock solutions were prepared; (#1) 3 mg PdCl_2_ was dissolved in 100 µl (170 mM) 6 M Gn^.^HCl buffer, pH 7. (#2) 5 mg DSF was dissolved in 100 µl (170 mM) ACN. (#3) 25 mg DTC was dissolved in 100 µl (1 M) H_2_O. (#4) 1 mg LiS was dissolved in 100 µl (222 mM) H_2_O. (#5) 1 mg glutathione (GSH) was dissolved in 100 µl (33 mM) H_2_O.

### α-conotoxin SI synthesis

The lyophilized conotoxin peptide (0.5 mg, 0.3 nmol) was dissolved in 670 µl (0.5 mM) 6 M Gn^.^HCl buffer, pH 7, and treated with 10 equiv. DSF (20 µl from stock) for 10 s at 37 °C. Subsequently, the pH of the reaction was adjusted to 1 using 0.1 M HCl and 15 equiv. PdCl_2_ (30 µl from stock #1) was added for 5 min at 37 °C. Then, 30 equiv. (20 µl from stock #3) DTC followed by 10 equiv. DSF were added. pH adjustment to 7 and incubation at 37 °C for 10 s immediately afforded the native α-conotoxin SI.

### Plectasin synthesis

The lyophilized plectasin peptide (0.5 mg, 0.1 nmol) was dissolved in 208 µl (0.5 mM) 6 M Gn^.^HCl buffer, pH 7, and treated with 10 equiv. DSF (6 µl from stock #2) for 10 s followed by exposure to UV irradiation at 350 nm for 8 min at room temperature. Subsequently the pH of the reaction was adjusted to 1 by 0.1 M HCl and 15 equiv. PdCl_2_ (9 µl from stock #1) was added for 5 min at 37 °C. Then, 30 equiv. (3 µl from stock #3) DTC was added followed by in situ addition of 2 equiv. LiS (1 µl from stock #4) and 10 equiv. DSF. pH adjustment to 7 and incubation at 37 °C for 10 s immediately afforded the native plectasin. The addition of 2 equiv. LiS was found to facilitate the recovery of the peptide from the bounded Pd residues.

### EETI-II synthesis

The lyophilized EETI-II peptide (0.5 mg, 0.1 nmol) was dissolved in 303 µl 6 M Gn^.^HCl buffer, pH 7 (0.5 mM), and treated with 10 equiv. DSF (9 µl from stock #2) was added for 10 s followed by exposure to UV irradiation at 350 nm for 8 min at room temperature. Subsequently the pH of the reaction was adjusted to 1 using 0.1 M HCl and 15 equiv. PdCl_2_ (13 µl from stock #1) was added for 5 min at 37 °C. Then, 30 equiv. (4 µl from stock) DTC followed by in situ addition of 10 equiv. DSF, pH adjustment to 7 and incubation at 37 °C for 10 s afforded the native EETI-II.

### Linaclotide synthesis

The lyophilized linaclotide peptide (0.5 mg, 0.2 nmol) was dissolved in 515 µl 6 M Gn^.^HCl buffer, pH 7, (0.5 mM) and treated with 10 equiv. DSF (15 µl from stock #2) for 10 s followed by exposure to UV irradiation at 350 nm for 8 min at room temperature. Subsequently the pH of the reaction was adjusted to 1 by 0.1 M HCl and 15 equiv. PdCl_2_ (23 µl from stock #1) was added for 5 min at 37 °C. Then, 30 equiv. of DTC (7 µl from stock #3) followed by 2 equiv. GSH 10 equiv. (16 µl from stock # 5) and 10 equiv. DSF were added in situ. pH adjustment to 7 and incubation at 37 °C for 10 s immediately afforded the native linaclotide. The addition of 2 equiv. of GSH was found to facilitate the recovery of the peptide from the bounded Pd residues.

### Reporting summary

Further information on research design is available in the Nature Research Reporting Summary linked to this article.

## Supplementary information

Supplementary Information

Reporting Summary

## Data Availability

The authors declare that the data supporting the findings of this study are available within the paper and its supplementary information files. All data are available from the corresponding author upon reasonable request.
