# Supplementary material for: General synthetic strategy for regioselective ultrafast formation of disulfide bonds in peptides and proteins
Source: Nat Commun. 2021 Feb 8;12:870. doi: 10.1038/s41467-021-21209-0 (PMC7870662; doi:10.1038/s41467-021-21209-0)
Supplement: Supplementary file 1 — Supplementary Information [file 41467_2021_21209_MOESM1_ESM.pdf]

Supplementary Information

General Synthetic Strategy for Regioselective Ultrafast Formation of Disulfide Bonds in Peptides and Proteins

Shay Laps[1], Fatima Atamleh[1], Guy Kamnesky[1], Hao Sun[1] and Ashraf Brik[1]*

[1]Schulich Faculty of Chemistry, Technion-Israel Institute of Technology, Haifa 3200008, Israel

*Correspondence: abrik@technion.ac.il

Supplementary Methods

General methods

SPPS was carried out manually in syringes, equipped with teflon filters, purchased from Torviq or by using an automated peptide synthesizer (CS336X, CSBIO). If not differently described, all reactions were carried out at room temperature. Analytical grade *N*,*N*-dimethylformamide (DMF) was purchased from Bio-Lab Ltd. Commercial reagents were used without further purification. Resins were purchased from Creosalus, protected amino acids were purchased from GL Biochem and activating reagents [(2-(1H-benzotriazol-1-yl)-1,1,3,3-tetramethyluronium hexafluorophosphate (HBTU), hydroxybenzotriazole (HOBt), [(6-chlorobenzotriazol-1- yl)oxy-(dimethylamino)methylidene]-dimethylazanium hexafluorophosphate (HCTU), 1-[Bis(dimethylamino)methylene]-1H-1,2,3-triazolo[4,5-b]pyridinium 3-oxid hexafluorophosphate (HATU)] were purchased from Luxembourg Bio Technologies. Sodium diethyldithiocarbamate trihydrate, cas no: 20624-25-3 was purchased from Merck. Tetraethylthiuram disulfide (disulfiram), cas no: 97-77-8 was purchased from Merck. α-Conotoxin SI was purchased from Alomone Labs. Human CCL5 (RANTES) yeast derived recombinant protein was purchased from kingfisher-biotech. Linaclotide was purchased from chemscene-chemical reagents for life science. Analytical HPLC was performed on a Thermo instrument (Dionex Ultimate 3000) using analytical columns Xbridge (waters, BEH300 C4, 3.5µm, 4.6 × 150 mm) and XSelect (waters, CSH C18, 3.5 µm, 4.6 × 150 mm) at a flow rate of 1.2 mL/min. Preparative HPLC was performed on a Waters instrument using XSelect C18 10µm 19 × 250 mm and semi-preparative HPLC was performed on a Thermo Scientific instrument (Spectra System SCM1000) using Jupiter C4 10 µm, 300 Å, 250 × 10 mm column, at a flow rate of 15 and 4 mL/min respectively. All synthetic products were purified by HPLC and characterized by mass spectrometry using LCQ Fleet Ion Trap (Thermo Scientific). All

calculated masses have been reported as an average isotope composition. Buffer A: 0.1% TFA in water; buffer B: 0.1% TFA in acetonitrile. Yields were determined in mg scale.

List of the protected amino acids used in peptides synthesis

Fmoc-Gly-OH, Fmoc-Ala-OH, Fmoc-Val-OH, Fmoc-Leu-OH, Fmoc-Ile-OH, Fmoc-Phe-OH, Fmoc-Pro-OH, Fmoc-His(Trt)-OH, Fmoc-Asn(Trt)-OH, Fmoc-Gln(Trt)-OH, Fmoc-Arg(Pbf)-OH, Fmoc-Lys(Boc)-OH, Fmoc-Tyr(tBu)-OH, Fmoc-Ser(tBu)-OH, Fmoc-Thr(tBu)-OH, Fmoc-Asp(OtBu)-OH, Fmoc-Glu(OtBu)-OH, Fmoc-Trp(Boc)-OH, Fmoc-Met-OH, Fmoc-Nle-OH, Fmoc-Cys(Acm)-OH, Fmoc-Cys(NBzl)-OH, Fmoc-Cys(Trt)-OH, Boc-Cys(Trt)-OH, Boc-Ser-OH, Boc-Gly-OH, Boc-Ile-OH, Fmoc-Asp(Boc)-Thr(ψMe,MePro)-OH, Fmoc-Asp(Boc)-Ser(ψMe,MePro)-OH, Fmoc-Tyr(Trt)-Thr(ψMe,MePro)-OH, Fmoc-Val-Thr(ψMe,MePro)-OH.

Entry	Peptide/protein	Sequence
1	α-conotoxin- SI	ICCNPACGPKYSC
2	RANTES	SPYSSDTTPCCFAYIARPLPRAHIKEYFYTSGKC SNPAVVFVTRKNRQVCANPEKKWVREYINSLEMS
3	Plectasin	GFGCNGPWDEDDNleQCHNHCKS IKGYKGGYCAKGGFVCKCY
4	Linaclotide	CCEYCCNPACTGCY
6	EETI-II	GCPRILNleRCKQDSDCLAGCVCGPNGFCG

Supplementary Table. 1 sequences of the prepared peptides and proteins.

Synthesis of protected α-conotoxin- SI linear peptide

The synthesis was carried out using Fmoc-SPPS on a Rink amide resin (0.26 mmol/g, 0.2 mmol scale). Peptide synthesis was performed on peptide synthesizer in presence of 4 equiv. of amino acid, HCTU and 8 equiv. of N,N'- diisopropylethylamine (DIEA).

The pre-swollen resin was treated with 20% piperidine in DMF containing 0.1 mmol HOBt (3-5-3 min) to remove the Fmoc-protecting group. Deprotection and cleavage from the resin: The resin was washed with DMF, MeOH, DCM and dried. The peptide was cleaved using trifluoroacetic acid (TFA):triisopropylsilane (TIS):water (95:2.5:2.5) cocktail for 2 h. The cleavage mixture was filtered and the combined filtrate was added dropwise to a 10-fold volume of cold ether and centrifuged. The precipitated crude peptide was dissolved in 50% acetonitrile/water and lyophilized. The HPLC analysis was carried out on a C18 analytical column using a gradient of 0-60% B over 30 min. For preparative HPLC, C18 column in gradient of 0-40% B was used to provide the peptide in ~50% yield.

Synthesis of protected RANTES linear peptide

The synthesis was carried out using Fmoc-SPPS on a Rink amide resin (0.26 mmol/g, 0.2 mmol scale). Peptide synthesis was performed on peptide synthesizer and cleavage from the resin as described above. Fmoc-Asp(Boc)-Thr(ψMe,MePro)-OH, Fmoc-Tyr(Trt)-Thr(ψMe,MePro)-OH and Fmoc-Val-Thr(ψMe,MePro)-OH was coupled manually using 2.5 equiv. AA/HATU and 5 equiv. DIEA for 2 h. The precipitated crude peptide was dissolved in 50% acetonitrile/water and lyophilized. The HPLC analysis was carried out on a C4 analytical column using a gradient of 0-60% B over 30 min. For preparative HPLC, C4 column in gradient of 20-60% B was used to provide the peptide in ~50% yield.

Synthesis of α-conotoxin- SI linear peptide bearing Cys(Acm) and Cys(NBzl)

The synthesis was carried out using Fmoc-SPPS on a Rink amide resin (0.26 mmol/g, 0.2 mmol scale). Peptide synthesis was performed on peptide synthesizer and cleavage from the resin as described above. Fmoc-Cys(NBzl)-OH was coupled manually using

2.5 equiv. AA/HATU and 5 equiv. DIEA for 2 h. The precipitated crude peptide was dissolved in 50% acetonitrile/water and lyophilized. The HPLC analysis was carried out on a C4 analytical column using a gradient of 10-35% B over 30 min. For preparative HPLC, C4 column in gradient of 20-60% B was used to provide the peptide in ~50% yield.

Synthesis of protected plectasin linear peptide

The synthesis was carried out using Fmoc-SPPS on a Rink amide resin (0.26 mmol/g, 0.2 mmol scale). Peptide synthesis was performed on peptide synthesizer and cleavage from the resin as described above. Fmoc-Cys(NBzl)-OH was coupled manually using 2.5 equiv. AA/HATU and 5 equiv. DIEA for 2 h. The precipitated crude peptide was dissolved in 50% acetonitrile/water and lyophilized. The HPLC analysis was carried out on a C4 analytical column using a gradient of 10-35% B over 30 min. For preparative HPLC, C4 column in gradient of 20-60% B was used to provide the peptide in ~50% yield.

Synthesis of unprotected plectasin linear peptide

The synthesis was carried out using Fmoc-SPPS on a Rink amide resin (0.26 mmol/g, 0.2 mmol scale). Peptide synthesis was performed on peptide synthesizer and cleavage from the resin as described above. The precipitated crude peptide was dissolved in 50% acetonitrile/water and lyophilized. The HPLC analysis was carried out on a C4 analytical column using a gradient of 0-60% B over 30 min. For preparative HPLC, C4 column in gradient of 20-60% B was used to provide the peptide in ~50% yield.

Oxidative folding of unprotected plectasin

The lyophilized linear peptide was dissolved in 6M guanidine hydrochloride buffer (200mM Na2HPO4) at 3 mg/mL concentration and rapidly diluted (6-fold) with a degassed solution of 100 mM tris-hydroxymethylaminomethane containing 9.2 mM L-

cysteine and 1.2 mM L-cystine hydrochloride at pH 8.4, to give a final concentration of 0.5 mg/mL peptide and 1M guanidine hydrochloride in the folding buffer. The folding was completed within 3 h at room temperature, as determined by analytical LC-MS.

Synthesis of protected EETI-II

The synthesis was carried out using Fmoc-SPPS on a Rink amide resin (0.26 mmol/g, 0.2 mmol scale). Peptide synthesis was performed on peptide synthesizer and cleavage from the resin as described above. Fmoc-Asp(Boc)-Ser(ψMe,MePro)-OH and Fmoc-Cys(NBzl)-OH was coupled manually using 2.5 equiv. AA/HATU and 5 equiv. DIEA for 2 h. The precipitated crude peptide was dissolved in 50% acetonitrile/water and lyophilized. The HPLC analysis was carried out on a C4 analytical column using a gradient of 10-35% B over 30 min. For preparative HPLC, C4 column in gradient of 20-60% B was used to provide the peptide in ~50% yield.

Synthesis of unprotected EETI-II

The synthesis was carried out using Fmoc-SPPS on Rink amide resin (0.26 mmol/g, 0.2 mmol scale). Peptide synthesis was performed on peptide synthesizer and cleavage from the resin as described above. Fmoc-Asp(Boc)-Ser(ψMe,MePro)-OH was coupled manually using 2.5 equiv. AA/HATU and 5 equiv. DIEA for 2 h. The precipitated crude peptide was dissolved in 50% acetonitrile/water and lyophilized. The HPLC analysis was carried out on a C4 analytical column using a gradient of 0-60% B over 30 min. For preparative HPLC, C4 column in gradient of 20-60% B was used to provide the peptide in ~50% yield.

Oxidative folding of unprotected EETI-II

Purified EETI-II was dissolved in sodium phosphate buffer (0.2 M, pH 8) and oxidized by stirring open to the atmosphere for 3 days.

Synthesis of protected linaclotide:

The synthesis was carried out using Fmoc-SPPS on 2-chlorotrityl chloride resin (0.26 mmol/g, 0.2 mmol scale). Peptide synthesis was performed on peptide synthesizer and cleavage from the resin as described above. Fmoc-Cys(NBzl)-OH was coupled manually using 2.5 equiv. AA/HATU and 5 equiv. DIEA for 2 h. The precipitated crude peptide was dissolved in 50% acetonitrile/water and lyophilized. The HPLC analysis was carried out on a C4 analytical column using a gradient of 0-60% B over 30 min. For preparative HPLC, C4 column in gradient of 20-60% B was used to provide the peptide in ~50% yield.

CD spectroscopy of purified α-conotoxin-SI:

The peptide was dissolved in sodium acetate buffer (0.35 mg/ml) at pH 7. The exact concentration of the protein solution was determined by Pierce® BCA Protein Assay Kit, Thermo scientific. With this solution circular dichroism spectrum was recorded in a Chirascan (Applied Photophysics) instrument.

CD spectroscopy of commercially available α-conotoxin-SI:

The peptide was dissolved in sodium acetate buffer (0.063 mg/ml) at pH 7. The exact concentration of the protein solution was determined by Pierce® BCA Protein Assay Kit, Thermo scientific. With this solution circular dichroism spectrum was recorded in a Chirascan (Applied Photophysics) instrument.

CD spectroscopy of purified RANTES:

The peptide was dissolved in phosphate-buffered saline (PBS) (1.58 mg/ml) at pH 7. The exact concentration of the protein solution was determined by Pierce® BCA Protein Assay Kit, Thermo scientific. With this solution circular dichroism spectrum was recorded in a Chirascan (Applied Photophysics) instrument.

CD spectroscopy of purified plectasin:

The peptide was dissolved in water (0.37mg/ml) at pH 7. The exact concentration of the protein solution was determined by Pierce® BCA Protein Assay Kit, Thermo scientific. The circular dichroism spectrum was recorded in a Chirascan (Applied Photophysics) instrument.

CD spectroscopy of purified EETI-II:

The peptide was dissolved in phosphate-buffered saline (PBS) (3.33 mg/ml) at pH 7. The exact concentration of the protein solution was determined by Pierce® BCA Protein Assay Kit, Thermo scientific. The circular dichroism spectrum was recorded in a Chirascan (Applied Photophysics) instrument.

Antimicrobial assay for plectasin:

Fresh overnight colonies of methicillin resistant staphylococcus aureus (MRSA) were suspended in LB medium. The diluted bacterial suspensions were added to wells containing serial two-fold dilutions of synthetic protein or trimethoprim (TMP). The polypropylene trays were incubated at 37 ^{o}C in ambient air for 7 h followed by absorbance readings at 600 nm to measure bacterial growth. Measurements were performed in triplicate.

Trypsin inhibition activity assay for EETI-II:

EETI-II in 67 mM phosphate buffer, pH 7.6 was incubated with trypsin (0.3 nmol) in 1 mM HCl for 5 minutes at 25 ^{o}C. The solution was then added to a solution of Na - benzoyl-L-arginine ethyl ester (0.25 mM BAEE, 67 mM phosphate, pH 7.6, 25 ^{o}C) to a total of 3 mL and the change in absorbance was monitored at 253 nm. Initial rates were determined by the change in absorbance over time, and these rates were used to calculate percent trypsin activity. Rate determinations were performed in triplicate.

Ultrafast one-pot two disulfide bonds formation in RANTES:

The lyophilized RANTES peptide (0.5 mg, 0.06 nmol) was dissolved in 125 µl (0.5 mM) 6 M Gn·HCl buffer, pH 7, and treated with 10 equiv. DSF (4 µl from stock) for 1 s at 37 ^{o}C. Subsequently, the pH of the reaction was adjusted to 1 by 0.1 M HCl and 15 equiv. $PdCl_2$ (6 µl from stock) was added for 5 min at 37 ^{o}C. Then, 30 equiv. (2 µl from stock) DTC followed by 10 equiv. were added. Immediately after the pH was readjusted to 7 and put in 37 ^{o}C for 1 s to afford the native RANTES.

Pd/UV light promoted one-pot two disulfide bonds formation:

The lyophilized α-conotoxin peptide (0.5 mg, 0.28 nmol) was dissolved in 565 µl (0.5 mM) 6 M Gn·HCl buffer, pH 7, and treated with 10 equiv. DSF (17 µl from stock) under UV irradiation at 350 nm for 8 min in room temperature. Subsequently the pH of the reaction was adjusted to 1 by 0.1 M HCl and 15 equiv. $PdCl_2$ (25 µl from stock) was added for 5 min at 37 ^{o}C. Then, 30 equiv. (8 µl from stock) DTC followed by 10 equiv. DSF were added. Immediately after the pH was readjusted to 7 and put in 37 ^{o}C for 1 s to afford the native α-conotoxin SI.

The following stock solutions were prepared; (#1) 3 mg $PdCl_2$ was dissolved in 100 µl (170 mM) 6 M Gn·HCl buffer, pH 7. (#2) 5 mg DSF was dissolved in 100 µl (170 mM) ACN. (#3) 25 mg DTC was dissolved in 100 µl (1 M) H_2O. (#4) 1 mg LiS was dissolved in 100 µl (222 mM) H_2O. (#5) 1 mg glutathione (GSH) was dissolved in 100 µl (33 mM) H_2O.

α-conotoxin SI synthesis. The lyophilized conotoxin peptide (0.5 mg, 0.3 nmol) was dissolved in 670 µl (0.5 mM) 6 M Gn·HCl buffer, pH 7, and treated with 10 equiv. DSF (20 µl from stock) for 10 s at 37 ^{o}C. Subsequently, the pH of the reaction was adjusted to 1 using 0.1 M HCl and 15 equiv. $PdCl_2$ (30 µl from stock #1) was added for 5 min at 37 ^{o}C. Then, 30 equiv. (20 µl from stock #3) DTC followed by 10 equiv. DSF were

added. pH adjustment to 7 and incubation at 37 ^{o}C for 10 s immediately afforded the native α-conotoxin SI.

Plectasin synthesis. The lyophilized plectasin peptide (0.5 mg, 0.1 nmol) was dissolved in 208 µl (0.5 mM) 6 M Gn·HCl buffer, pH 7, and treated with 10 equiv. DSF (6 µl from stock #2) for 10 s followed by exposure to UV irradiation at 350 nm for 8 min at room temperature. Subsequently the pH of the reaction was adjusted to 1 by 0.1 M HCl and 15 equiv. $PdCl_2$ (9 µl from stock #1) was added for 5 min at 37 ^{o}C. Then, 30 equiv. (3 µl from stock #3) DTC was added followed by in situ addition of 2 equiv. LiS (1 µl from stock #4) and 10 equiv. DSF. pH adjustment to 7 and incubation at 37 ^{o}C for 10 s immediately afforded the native plectasin. The addition of 2 equiv. LiS was found to facilitate the recovery of the peptide from the bound Pd residues.

EETI-II Synthesis. The lyophilized EETI-II peptide (0.5 mg, 0.1 nmol) was dissolved in 303 µl 6 M Gn·HCl buffer, pH 7 (0.5 mM), and treated with 10 equiv. DSF (9 µl from stock #2) was added for 10 s followed by exposure to UV irradiation at 350 nm for 8 min at room temperature. Subsequently the pH of the reaction was adjusted to 1 using 0.1 M HCl and 15 equiv. $PdCl_2$ (13 µl from stock #1) was added for 5 min at 37 ^{o}C. Then, 30 equiv. (4 µl from stock) DTC followed by in situ addition of 10 equiv. DSF, pH adjustment to 7 and incubation at 37 ^{o}C for 10 s afforded the native EETI-II.

Linaclotide synthesis. The lyophilized linaclotide peptide (0.5 mg, 0.2 nmol) was dissolved in 515 µl 6 M Gn·HCl buffer, pH 7, (0.5 mM) and treated with 10 equiv. DSF (15 µl from stock #2) for 10 s followed by exposure to UV irradiation at 350 nm for 8 min at room temperature. Subsequently the pH of the reaction was adjusted to 1 by 0.1 M HCl and 15 equiv. $PdCl_2$ (23 µl from stock #1) was added for 5 min at 37 ^{o}C. Then, 30 equiv. of DTC (7 µl from stock #3) followed by 2 equiv. GSH 10 equiv. (16

μl from stock # 5) and 10 equiv. DSF were added in situ. pH adjustment to 7 and incubation at 37 °C for 10 s immediately afforded the native linaclotide. The addition of 2 equiv. of GSH was found to facilitate the recovery of the peptide from the bounded Pd residues.

Supplementary Figures

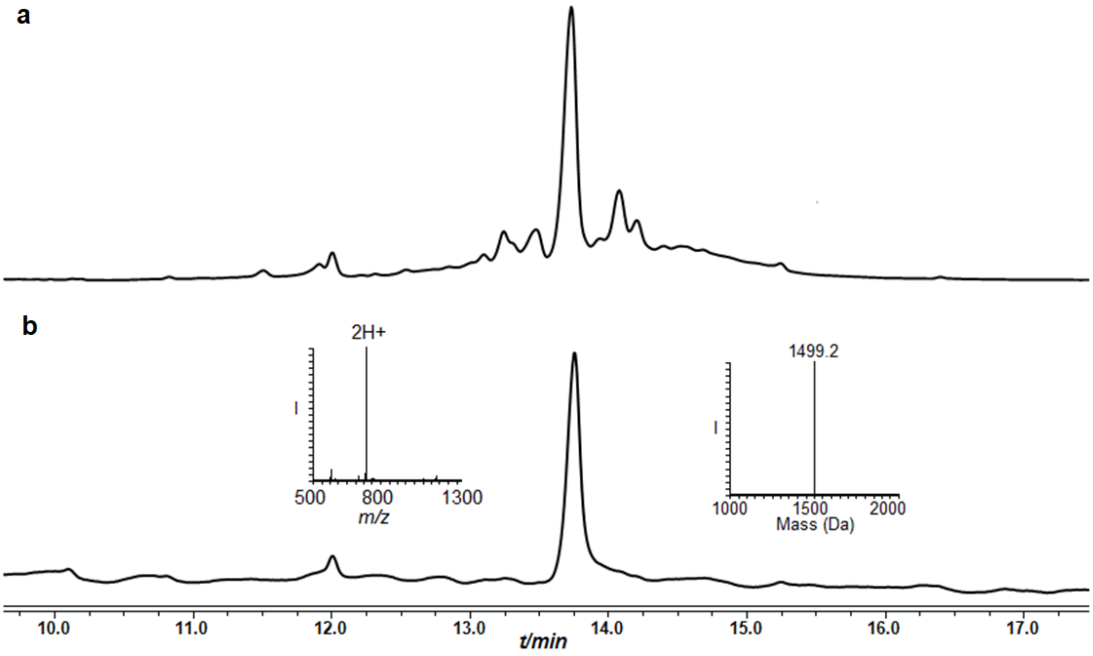

Supplementary Fig. 1 SPPS of α-conotoxin SI modified with two Acm PGs at Cys (2&7). HPLC and mass analyses of (**a**) crude: the main peak corresponds to the desired peptide (**b**) purified: the main peak corresponds to the desired peptide, with the observed mass 1499± 0.2 Da (calcd 1499.2 Da, average isotopes).

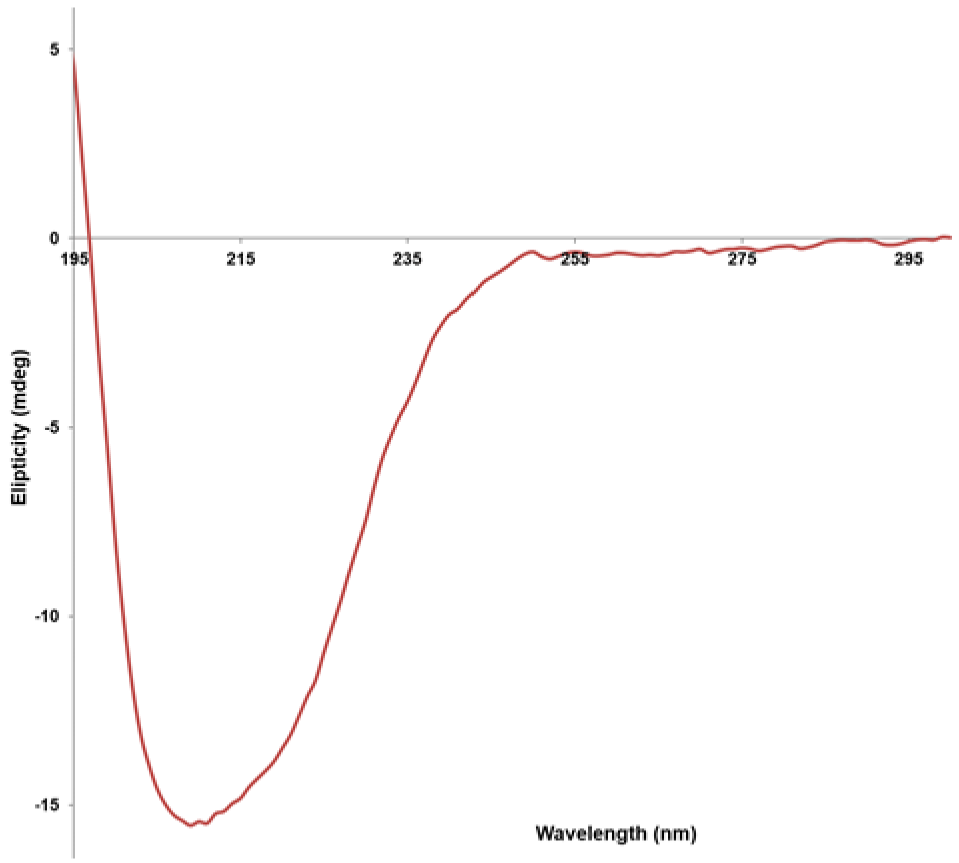

Supplementary Fig. 2 CD for α-conotoxin SI commercially available product.

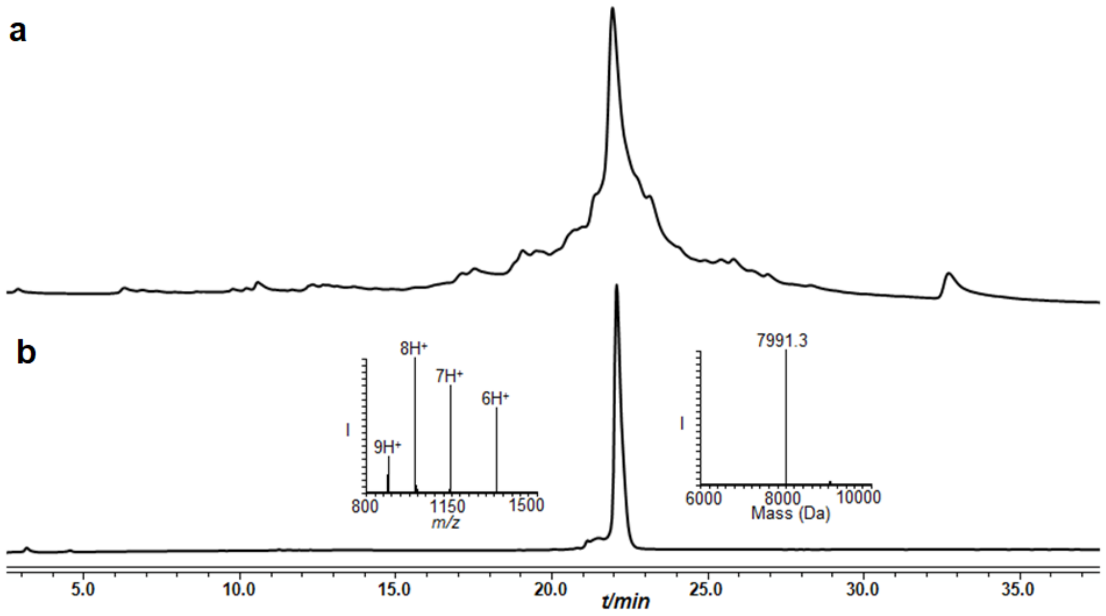

Supplementary Fig. 3 SPPS of RANTES modified with two Acm PGs at Cys (11&50). HPLC and mass analyses of (**a**) crude: the main peak corresponds to the desired peptide (**b**) purified: the main peak corresponds to the desired peptide, with the observed mass 7991.3 ± 0.1 Da, calcd 7992.0 Da (average isotopes).

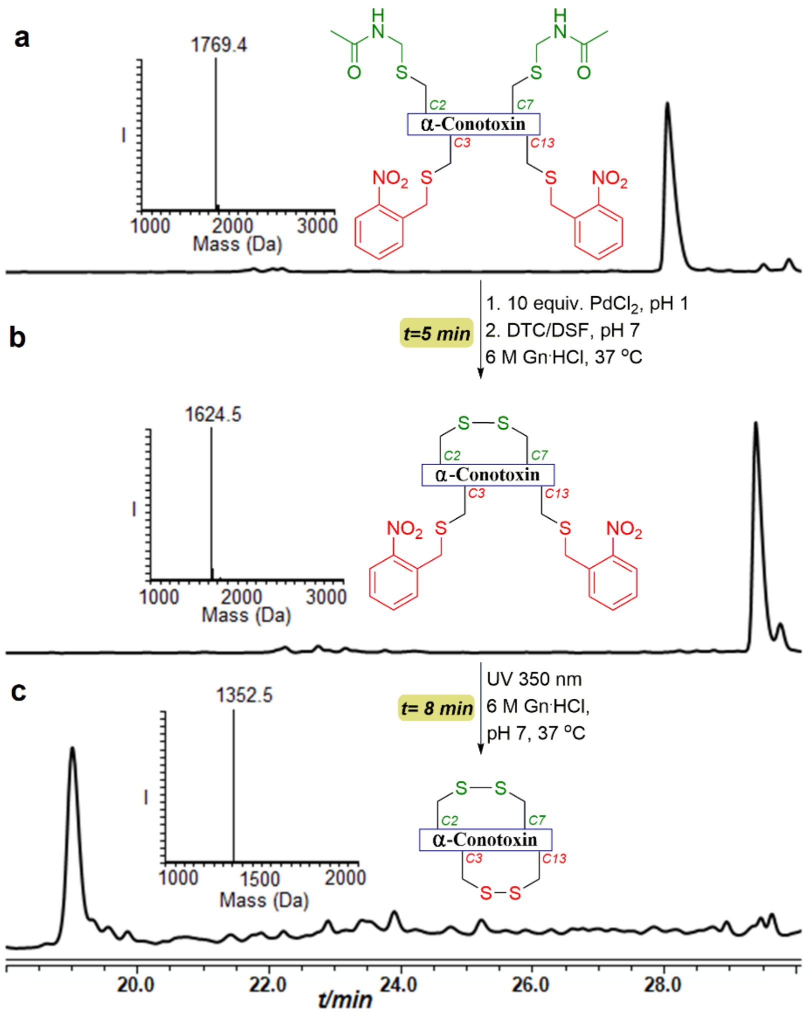

Supplementary Fig. 4 α-conotoxin synthesis via Pd/UV chemoselective decaging. (**a**) HPLC-ESI MS analyses: Reaction at time zero, the main peak corresponds to α-conotoxin peptide modified with two Acm groups at Cys (2&7) and NBzl at Cys (3&13) with the observed mass 1769.4 ± 0.1 Da, calcd 1769.6 Da (average isotopes). (**b**) Reaction after 5 min: the main peak corresponds to α-conotoxin peptide bearing one disulfide bond modified with two NBzl groups at Cys (3&13) with the observed mass 1624.5 ± 0.1 Da, calcd 1625.6 Da (average isotopes). (**c**) Reaction after 13 min: the main peak corresponds to α-conotoxin peptide bearing two disulfide bonds with the observed mass 1352.5 ± 0.1 Da, calcd 1353.6 Da (average isotopes).

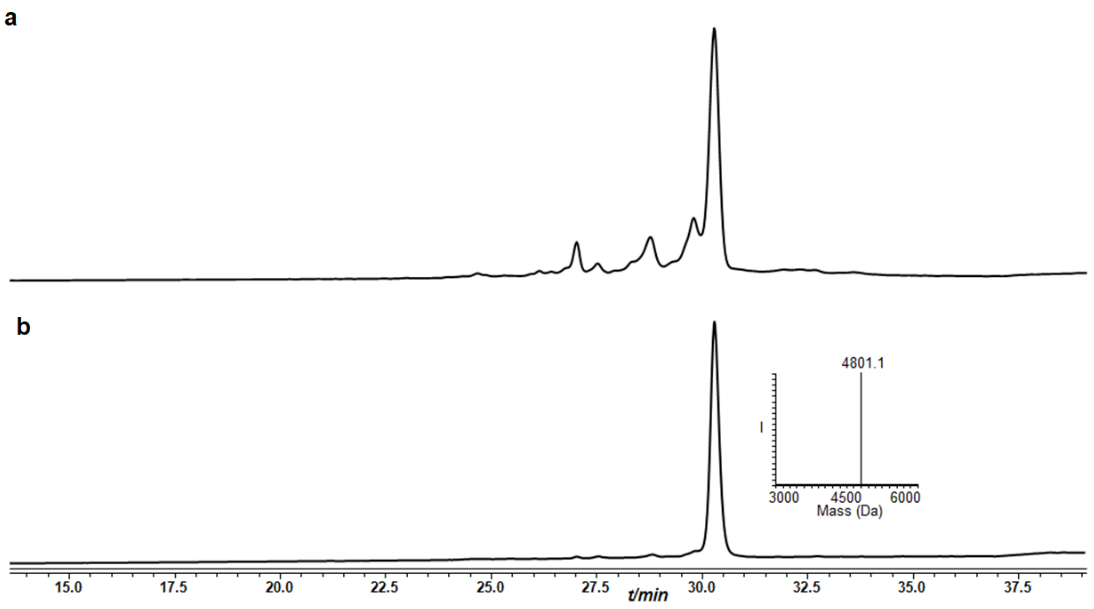

Supplementary Fig. 5 SPPS of plectasin modified with two NBzl at Cys (19&39) and two Acm PGs at Cys (15&37). HPLC and mass analyses of (**a**) crude: the main peak corresponds to the desired peptide (**b**) purified: the main peak corresponds to the desired peptide, with the observed mass 4801.1± 0.2 Da (calcd 4801.0 Da, average isotopes).

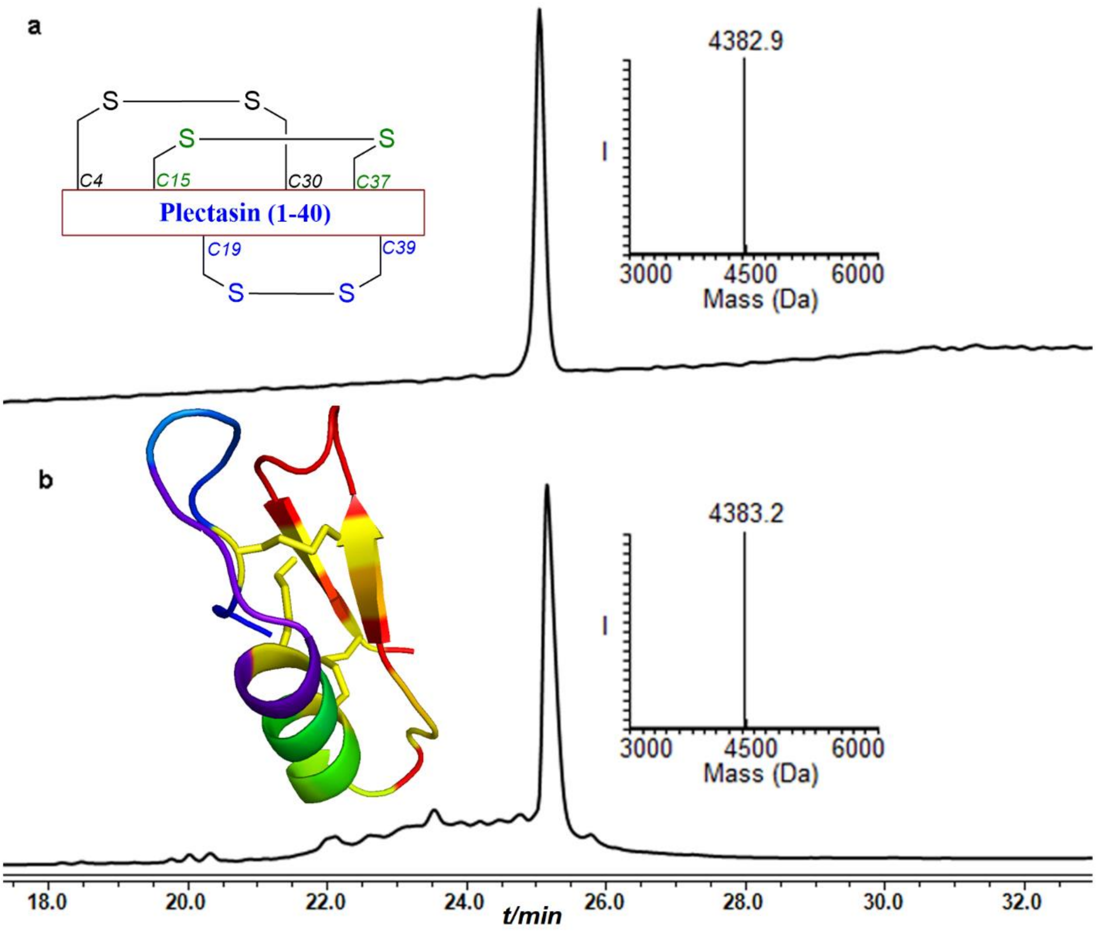

Supplementary Fig. 6 Plectasin chromatographic comparison of product obtained by our de novo synthetic strategy and with the oxidative folding product. (**a**) HPLC-ESI MS analyses of purified synthetic plectasin with the observed mass 4382.9 ± 0.1 Da, calcd 4383.0 Da (average isotopes). (**b**) HPLC-ESI MS analyses of oxidative folding product plectasin with the observed mass 4383.2 ± 0.1 Da, calcd 4383.0 Da (average isotopes).

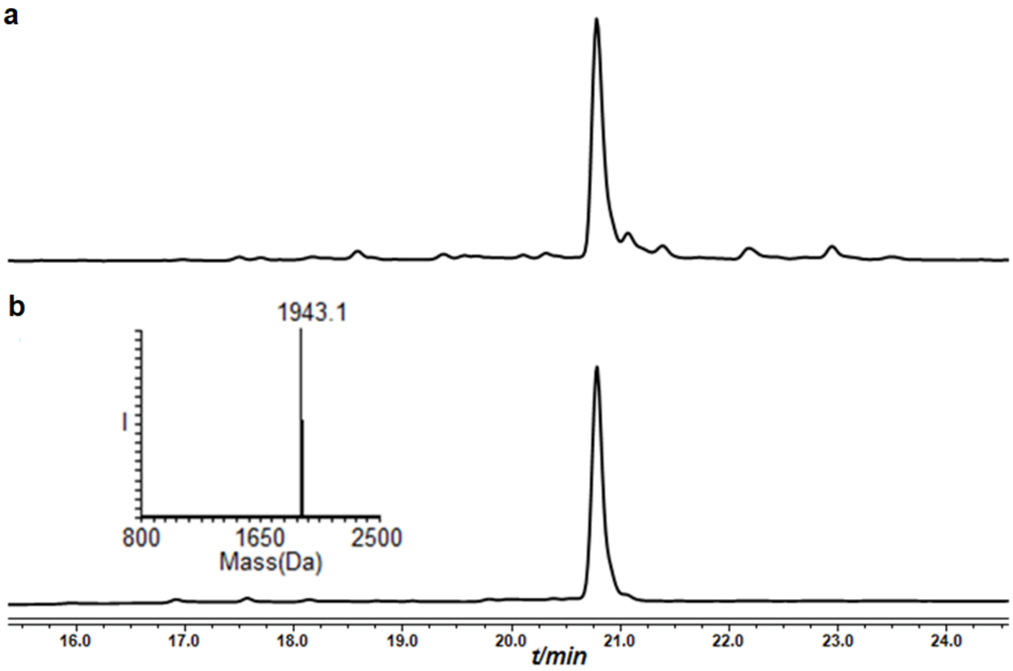

Supplementary Fig. 7 SPPS of linaclotide modified with two NBzl PGs at Cys (2&10) and two Acm PGs at Cys (1&6). HPLC and mass analyses of (**a**) crude: the main peak corresponds to the desired peptide (**b**) purified: the main peak corresponds to the desired peptide, with the observed mass 1943.1± 0.2 Da (calcd 1944.2 Da, average isotopes).

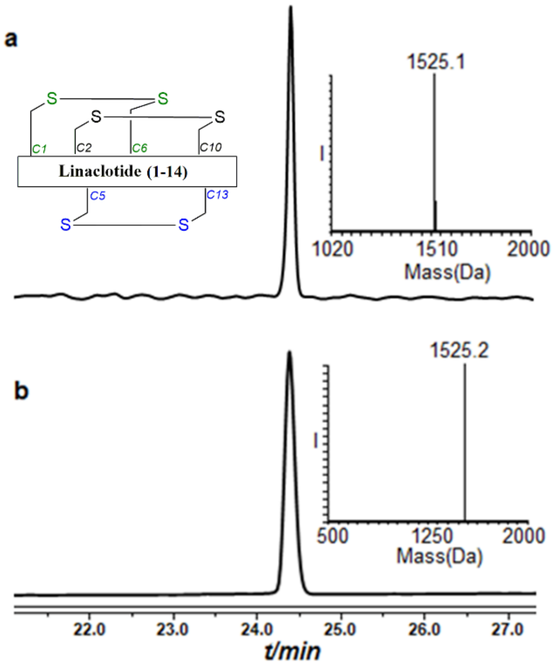

Supplementary Fig. 8 Linaclotide chromatographic comparison with a commercially available product. (**a**) The peak corresponds to the purified product with the three disulfides bonds with the observed mass1525.1± 0.3 Da (calcd 1525.2 Da, average isotopes). (**b**) The peak corresponds to the commercially available Linaclotide, with the observed mass 1525.2± 0.3 Da (calcd 1525.2 Da, average isotopes).

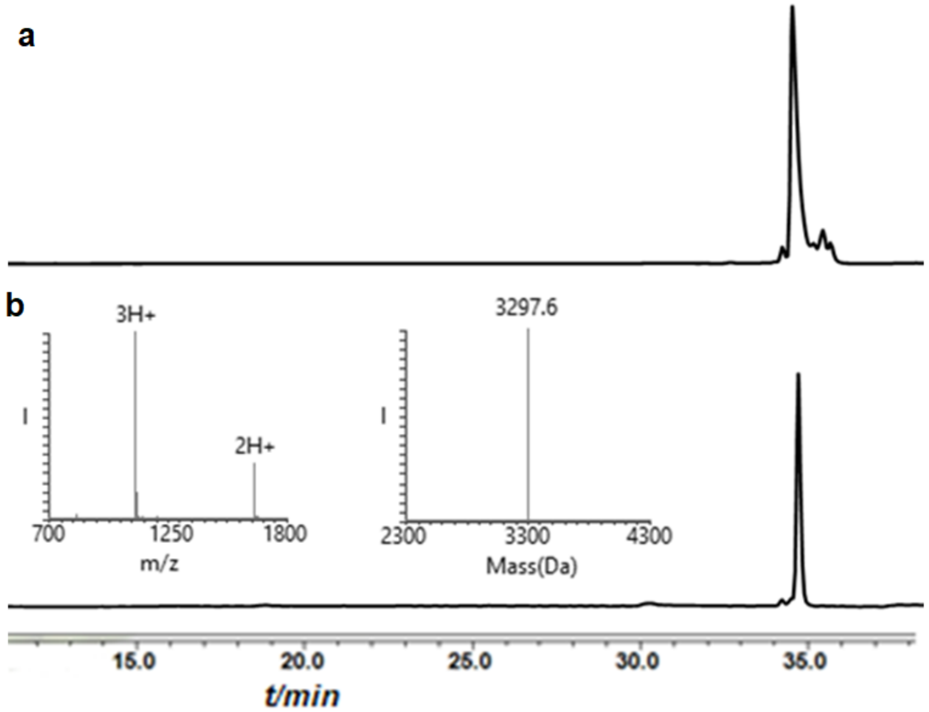

Supplementary Fig. 9 SPPS of EETI-II modified with two NBzl at Cys (15&27) and two Acm PGs at Cys (2&19). HPLC and mass analyses of (**a**) crude: the main

peak corresponds to the desired peptide (**b**) purified: the main peak corresponds to the desired peptide, with the observed mass 3297.6 ± 0.2 Da (calcd 3297.4 Da, average isotopes).

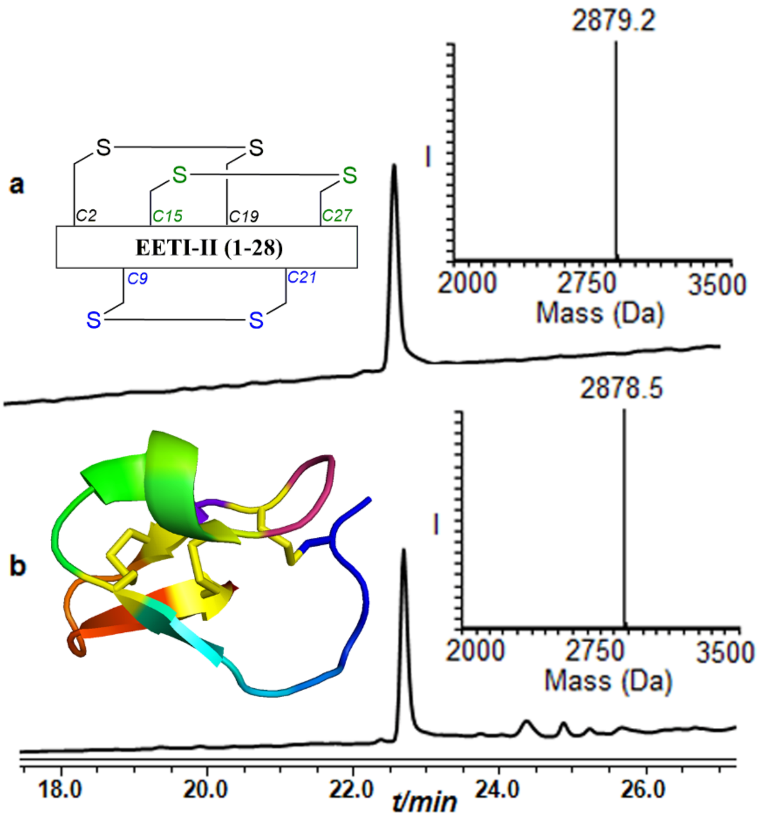

Supplementary Fig. 10 EETI-II chromatographic comparison. HPLC and Mass analyses of the (**a**) product obtained by our de novo synthetic strategy with the three disulfides bonds with the observed mass 2879.2± 0.6 Da (calcd 2879.3, average isotopes). (**b**) and with the oxidative folding product with the observed mass 2878.5± 0.6 Da (calcd 2878.3, average isotopes).
